# Endophytic microbiota and ectomycorrhizal structure of *Alnus* *glutinosa* Gaertn. at saline and nonsaline forest sites

**DOI:** 10.1038/s41598-023-49447-w

**Published:** 2023-12-20

**Authors:** Dominika Thiem, Marc Goebel, Marcin Gołębiewski, Christel Baum, Piotr Koczorski, Sonia Szymańska, Katarzyna Hrynkiewicz

**Affiliations:** 1 Department of Microbiology, Faculty of Biological and Veterinary Sciences, Nicolaus Copernicus University (NCU), Lwowska 1, 87-100 Torun, Poland; 2 Centre of Modern Interdisciplinary Technologies, NCU, Wilenska 4, 87-100 Torun, Poland; 3 Department of Natural Resources and the Environment, Cornell University, 111 Fernow Hall, Ithaca, NY 14853 USA; 4 Chair of Plant Physiology and Biotechnology, Faculty of Biological and Veterinary Sciences, NCU, Lwowska 1, 87-100 Torun, Poland; 5 Soil Science, University of Rostock, Justus-von-Liebig-Weg 6, 18059 Rostock, Germany

**Keywords:** Microbial communities, Metagenomics, Microbial ecology, Microbiome, Symbiosis, Soil microbiology

## Abstract

The tolerance of European alder (*Alnus* *glutinosa* Gaertn.) to soil salinity can be attributed to symbiosis with microorganisms at the absorptive root level. However, it is uncertain how soil salinity impacts microbial recruitment in the following growing season. We describe the bacterial and fungal communities in the rhizosphere and endosphere of *A.* *glutinosa* absorptive roots at three tested sites with different salinity level. We determined the morphological diversity of ectomycorrhizal (ECM) fungi, the endophytic microbiota in the rhizosphere, and the colonization of new absorptive roots in the following growing season. While bacterial diversity in the rhizosphere was higher than that in the absorptive root endosphere, the opposite was true for fungi. Actinomycetota, Frankiales, *Acidothermus* sp. and *Streptomyces* sp. were more abundant in the endosphere than in the rhizosphere, while Actinomycetota and *Acidothermus* sp. dominated at saline sites compared to nonsaline sites. Basidiomycota, Thelephorales, Russulales, Helotiales, *Cortinarius* spp. and *Lactarius* spp. dominated the endosphere, while Ascomycota, Hypocreales and *Giberella* spp. dominated the rhizosphere. The ECM symbioses formed by Thelephorales (*Thelephora*, *Tomentella* spp.) constituted the core community with absorptive roots in the spring and further colonized new root tips during the growing season. With an increase in soil salinity, the overall fungal abundance decreased, and *Russula* spp. and *Cortinarius* spp. were not present at all. Similarly, salinity also negatively affected the average length of the absorptive root. In conclusion, the endophytic microbiota in the rhizosphere of *A.* *glutinosa* was driven by salinity and season, while the ECM morphotype community was determined by the soil fungal community present during the growing season and renewed in the spring.

## Introduction

Individuals of the genus *Alnus* are established around the globe^1,2^ and cope with abiotic stresses such as salinity^3^, flooding^2^ and drought^4^. The ability of *Alnus* species to form a tripartite symbiosis with ectomycorrhizal (ECM) and arbuscular mycorrhizal (AM) fungi, as well as nitrogen-fixing bacteria *Frankia* spp.^5^, which allows its establishment in soils with the presence of different stressors. Currently, approximately 30 ECM species^6^ and a few AM fungi (*Glomeraceae* or *Claroideoglomeraceae* family)^7^ have been identified, permitting the observation of the root endophyte community dynamics associated with different abiotic stressors over time.

Soil salinity, one of the most severe abiotic and persistent stressor, limits overall plant growth and decreases metabolic activity, especially in a considerable number of soil microorganisms^8^. Around the world, over 424 million hectares of topsoil (0–30 cm) and 833 million hectares of subsoil (30–100 cm) are affected by salinity^9^. In addition, the impact of global climate change results in increased soil salinity, exacerbated by extended periods of drought^10^. Both existing soil salinity and drought conditions act on symbionts and other endophytic microorganisms, which commonly facilitate host plant growth and survival during periods of environmental stress^11^. In our previous studies, we found that bacterial and fungal community structure in the roots of *Alnus* adjusted to specific levels of salinity, increasing or decreasing in diversity and quantity^12^. However, we did not find significant differences in bacterial and fungal communities in association with *Alnus* *glutinosa* Gaertn. roots during the growing season. We also expect the diversity and quantity of root microbiota to be stable during the winter season; however, we assume that certain microbial species overwinter and actively contribute to the continuous formation of symbiosis in the following spring.

In recent years, advances in metagenomic analyses have allowed us to gain a better understanding of microbial population dynamics via the analysis of DNA acquired directly from environmental samples^13^. However, metagenomic techniques do not allow us to distinguish between active and dormant microorganisms or even DNA from dead cells of microorganisms forming associations with plants, e.g., mycorrhizal symbiosis. Therefore, we decided to supplement DNA sequencing with the visual observation of seasonal growth belowground, using in situ minirhizotrons (MRs) to determine the growth dynamics of absorptive roots over time^14^ and the formation of ectomycorrhizal morphological structures of ECM fungi over time^15,16^. Observation of interactions between the bacterial and fungal rhizosphere and root communities in salinity conditions is particularly important in the context of searching for potential bioinoculates, including mycorrhizal ones. With the parallel application of the two techniques, DNA sequencing and visual MR observations of belowground growth dynamics in the presence of soil salinity, our objectives are (a) to describe the bacterial and fungal communities present in the rhizosphere and in absorptive fine roots of *A.* *glutinosa* under nonsaline and saline growth conditions after and before a growing season (fall of a year and spring of the following year), (b) to determine which fungi persist over winter and form symbioses with absorptive roots in spring of the following growing season, (c) to quantify the growth of absorptive root tips in soils with different levels of salinity, and (d) to determine the approximate morphological diversity of ECM fungi.

We hypothesize that (i) bacterial alpha-diversity will be higher in the rhizosphere than in absorptive roots, but overall, there will be no difference between seasons (fall and spring), both for bacteria and fungi, (ii) the salinity level and/or season (fall and spring) will determine the microbial community structure in the rhizosphere and in absorptive roots (endosphere), as well as elongation growth dynamics of absorptive roots, and (iii) a core of ECM fungi will be observed throughout the growing seasons (spring, summer, fall); however, the abundance would be negatively affected by salinity.

## Methods

### Site and soil description and environmental conditions

All three *A.* *glutinosa* species-specific forest sites are in geographical proximity in the forest district of Szubin in central-northern Poland and range in age from 23 to 26 years. The soils at the three sites are periodically waterlogged, have shallow horizons (20–30 cm thick) and are rich in highly decomposed organic matter (TOC 3.7–9.9%) with overlying sands^12,17^. According to the WRB classification (IUSS Working Group 2022), the soils can be described as Eutric Mollic Gleysols (Arenic, Hyperhumic). The nonsaline (NS) control site is located near Pszczółczyn (53°00′22.9″ N, 17°54′57.2″ E), and the two saline sites (SL-with lower salinity level and SH-with higher salinity level) are near Słonawy (53°01′26.6″ N, 17°37′47.3″ E). The salinity levels at the SL (lower salinity) and SH (higher salinity) sites originate from the Zechstein salt deposits rising to the soil surface, a process documented in this region since the Middle Ages^17^.

Levels of salinity (EC_e_) increased by site (NS < SL < SH) and ranged between 21.7 and 54.0 μS × cm^−1^ during two seasons (fall and spring) at the NS site and varied between 45.5 and 86.7 μS × cm^−1^ at the SL site and 146.7 to 389 μS × cm^−1^ at the SH site (Supplementary material, Table 1). According to the adopted salinity classes in electrical conductivity presented saline soils can be classified to slightly saline. Higher soil moisture in the spring significantly decreased EC_e_ levels at all sites. The level of sodium ions was positively correlated with salinity levels (NS < SL < SH) but was only significantly different between the control and saline sites in the previous fall. Due to the geological location of the tested sites the high levels of heavy metals ions were not suspected in the soil. Total organic carbon (%) and total nitrogen (%) were significantly higher at the NS site than at the two saline sites in fall and similar for NS and SH in spring, while soil pH was significantly higher for SL than for the other two sites (Supplementary material, Table 1).

In the forest district of Szubin, the total annual precipitation was lower in 2018 than in the two previous years, 432 mm vs. 767 mm (2016) and 646 mm (2017), respectively (Supplementary material, Fig. 1). In the spring of 2016 and 2017 (April-June), the average precipitation was 186 mm for each year, while it was 50% less (66 mm) in 2018. During the summer months (July–September), the average precipitation in 2016, 2017 and 2018 was 174 mm, 290 mm, and 161 mm, respectively. Air temperatures during the growing season (April-October) were relatively constant for the last 5 years (2014–2015) averaging between 14 and 16 °C and reached maximum temperatures of 22 °C in July/August (weather stations: Bydgoszcz, No. 253180220, and Chrząstwo, No. 253170210, Institute of Meteorology and Water Management, Warsaw, Poland).

### Soil and absorptive root sampling

At each of the three forest sites, we collected a soil cube (24 × 24 × 24 cm) at a 40 cm horizontal distance from five individual tree stems in October 2017 (fall) and in April 2018 (spring). Subsamples of the soil were collected for physicochemical soil properties (5 soil samples at each of the 3 sites, n = 15, both in the fall and spring), and absorptive roots (< 1 mm in diameter) were collected from the soil samples for metagenomic DNA isolation (5 soil samples at each of the 3 sites, n = 15, in both the fall and spring; S—rhizosphere and R—absorptive root endosphere samples). All the root samples were collected in accordance with institutional, national, and international guidelines and legislation with the permission of the forest district of Szubin.

In October 2017, we also installed acrylic minirhizotron tubes (MR, CID Bio-Science Inc.) at a 30° angle from the soil surface with a gas-powered auger (Harbour Freight Predator) at the individual trees from the locations where the soil cubes were collected (workflow of all experiments, Supplementary material, Fig. 2).

### Physicochemical analysis of soil

The soil moisture content was measured according to a previously described procedure^18^. Soil subsamples were air dried at room temperature (48 h) and sieved (5 mm size) before physicochemical properties were analysed, e.g., EC_e_ (electrical conductance in saturation extract, µS × cm^−1^), pH in calcium chloride (CaCl_2_), total organic carbon (TOC, %), total nitrogen (TN, %), phosphorus (P, mg/kg) and concentration of ions (calcium, magnesium, potassium, sodium (mg/g)). The statistical significance of each soil parameter was tested by Kruskal‒Wallis and with Dunn’s test as post hoc comparisons (PAST, ver. 4.03). All statistical tests utilized an alpha error at the 0.05 level.

### Marker gene amplicon analysis

Absorptive roots were gently separated from the soil and washed three times with tap water. All subsequent steps were performed under sterile conditions. Two grams of root material was surface sterilized in two steps, first using 70% ethanol for 30 s and then in 15% H_2_O_2_ for 2 min. Between the sterilization steps, the plant material was washed three times in distilled water. Water obtained after the last washing was spread on Petri plates with nutrient agar to assess the sterilization efficacy (3 replicates per variant)^19^. Only sterile roots were used in further analysis. Samples of soils and sterilized roots were lyophilized. Next, 50 mg of lyophilized soil or roots were weighed and put into reinforced tubes containing 100 mg of sterile quartz sand or sterile metal beads, respectively.

The isolation of total DNA was performed using the Power Soil DNA isolation Kit (MO BIO Laboratories, USA) according to the manufacturer's protocol. The obtained DNA was measured spectrophotometrically (Nanodrop 2000) and diluted to 1 ng/µl. Next, two types of libraries were prepared: bacterial (16S rRNA gene) and fungal (ITS region). The libraries were generated according to the procedure described by Thiem et al.^12^. Briefly, V3-V4 rRNA gene fragments and ITS1 regions were amplified using specific primers tagged with M13/M13R universal sequences. All PCR products were checked on 1.5% agarose gels in 1xTBE and then purified using Ampure (Beckman Coulter) according to the manufacturer’s protocol. PCR product concentrations were measured spectrophotometrically (Nanodrop), and the products were diluted to 4 ng/µl and used in a second round of PCR to introduce indices and P5/P7 adapters^12^. After purification and fluorimetric quantification, the quality of the pooled libraries was assessed on a Bioanalyzer chip (Agilent). The final pool was diluted to 4 nM, denatured, mixed with 5% of the PhiX control library and sequenced using a 2 × 300 cycles kit v.3 on a MiSeq machine (Illumina). Sequencing was performed using HPLC-purified versions of forward and reverse primers as well as reverse-complement of the reverse primer.

### Observations of ECM morphotypes and absorptive roots

For the two soil depth of 0–12 cm (L1) and 12–24 cm (L2), we identified the number of visible ECM morphotypes on absorptive root tips based on macroscopic characteristics and colour of the fungal mantle (according to features presented in the DEEMy database, http://www.deemy.de/).

Minirhizotron images were recorded with a CI-602 narrow gauge root imager (CID Bio-Science Inc., USA) during the growing season of 2018 and categorized into three seasons: spring (April–June), summer (July–September), and fall (October). Belowground growth dynamics of absorptive roots of *A.* *glutinosa* with their specific red, pointy root tips (root diameter ≥ 0.25 mm and < 1 mm) were captured at a vertical soil depth of 0–12 cm with a maximum image resolution of 1200 dpi. Individually collected MR images were enhanced by adjusting the exposure and contrast with GIMP 2.10 (GNU Image Manipulation Software)^20^ for better differentiation of absorptive roots on the variable colouration of the soil background. A subsection of each MR image was analysed to estimate the average number of absorptive roots in an MR image and scaled up to the full size of the MR image to estimate the accumulative number of absorptive root tips and elongation growth using WinRhizoTron 2020a (Regents Instruments Inc., Canada).

### Bioinformatic and statistical analyses

The sequencing reads were denoised and merged with dada2 as described in Koczorski et al.^22^. Then, fungal sequences were processed with ITSx^21^. The sequences were dereplicated, and OTUs were constructed using mothur v.1.39^23^ at a 0.03 dissimilarity level. Then, singletons as well as doubletons (OTUs consisting of one or two sequences only) were removed. The sequences were classified with a naive Bayesian classifier (minimum 80% bootstrap support was needed)^24^ using the SILVA Seed v.132 database for bacterial sequences and ITS1 extracted from UNITE v.8.2 (fungi) for fungal sequences. The final data were subsampled to 4500 (bacteria) and 1000 (fungi) sequences per sample 100 times, and average numbers of sequences for each OTU rounded to the nearest integer were used in downstream analyses. 

The significance of differences in means (number of observed OTUs, Shannon’s H’, Shannon’s E, group distribution between sites, seasons and source of isolation) was assessed with ANOVA and post hoc Tukey’s HSD, unless assumptions of normality of data and/or homogeneity of variance were violated, in which case robust ANOVA implemented in function raov of the Rfit package was used to check for general p value. To check significant physicochemical parameter effects on endophyte communities in soil and roots, nonmetric multidimensional scaling (NMDS) and canonical correspondence analysis (CCA) analyses were performed within R with vegan’s metaMDS and CCA functions. In the case of CCA, the forward selection procedure implemented in ordistep was used for model building. The significance of differences between sample clusters was assessed with ANOSIM and PERMANOVA in vegan’s anosim and adonis functions, respectively. The significance of CCA models was assessed with a permutational test (anova.cca). Variance partitioning was performed with the varpart function. Kruskal‒Wallis tests were performed to check the differences in the relative abundance of bacterial and fungal taxa in absorptive roots and the rhizosphere at three sites (NS, SL, SH) and two seasons (fall, spring).

The impact of soil salinity on the average growth of absorptive root tips and average absorptive root tip length were tested with fixed effects: site (NS, SL and SH) and growing season (spring, summer, fall) and the interaction (site x season) with a linear mixed effect model (JMP, SAS Institute, Cary, NC, USA). Minirhizotron tubes were coded as a random effect. If the assumption of normality was not met, the data were log-transformed. All figures were plotted with standard R graphic functions and SigmaPlot 14.

## Results

### Alpha diversity at three tested sites in fall and spring

**Figure 1:**
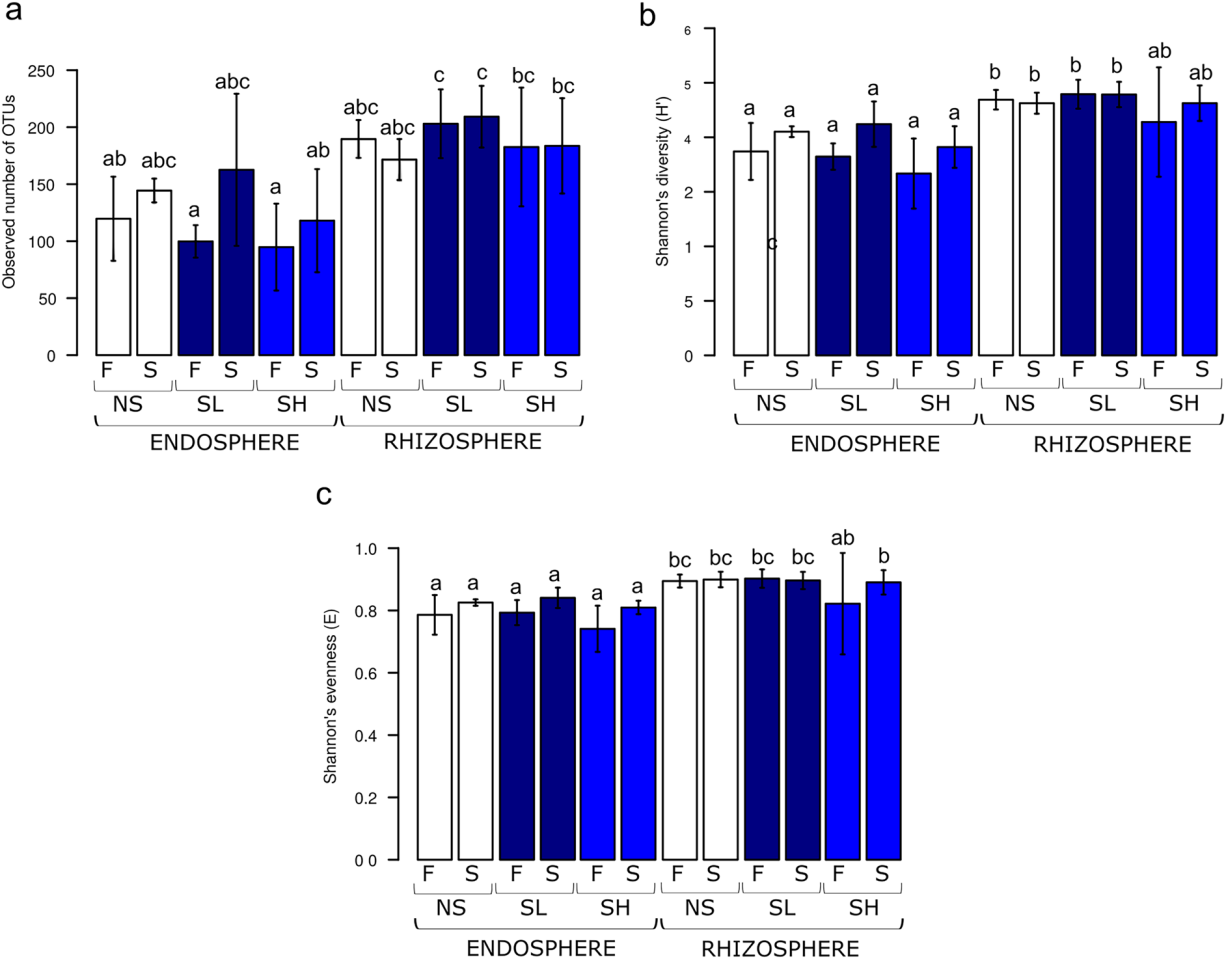
Alpha diversity of bacterial OTUs in rhizosphere and root endosphere of *A.* *glutinosa* of the three sites (*NS* nonsaline, *SL* saline with lower salinity, *SH* saline with higher salinity) in two seasons (*F* fall, *S* spring) presented as (**a**) observed number of OTUs, (**b**) Shannon’s H and (**c**) Shannon’s E. Robust ANOVA test with the Tukey’s post hoc analysis were used to assess significance of differences between variants for observed number of OTUs, KruskalWallis test with the Dunn post hoc comparisons were used in case of H’ and E indices. Variants labelled with the same letter are not significantly different (p ≤ 0.05).

Bacterial communities showed a higher diversity in the rhizosphere than in the root endosphere. In the fall, the rhizosphere had the highest numbers of bacterial OTUs at the saline sites (SL and SH), which differed significantly compared to the low OTU values in the endosphere (Fig. 1a). In the fall and spring, both indices of bacterial richness (Shannon H’) and evenness (Shannon E) were significantly higher in the rhizosphere than in the endosphere at the NS and SL sites (Fig. 1b, c). However, the SH site was not significantly different because of its high variability in index values.

**Figure 2:**
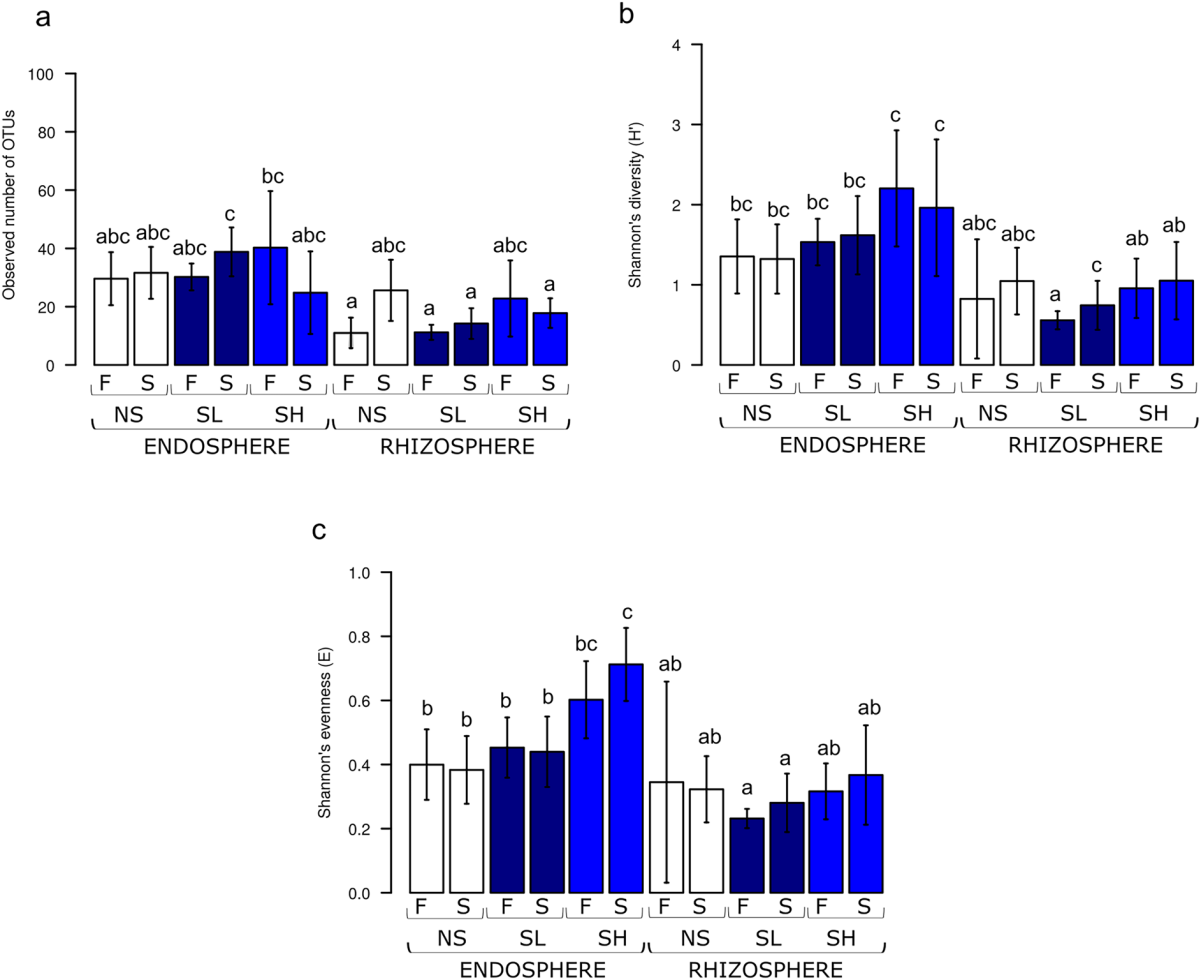
Alpha diversity of fungal OTUs in rhizosphere and root endosphere of *A.* *glutinosa* of the three sites (*NS* nonsaline, *SL* saline with lower salinity, *SH* saline with higher salinity) in two seasons (*F* fall, *S* spring) presented as (**a**) observed number of OTUs, (**b**) Shannon’s H and (**c**) Shannon’s E. Robust ANOVA test with Tukey’s post hoc analysis were used to assess significance of differences between variants for observed number of OTUs, Kruskal‒Wallis test with Dunn post hoc comparisons were used in case of H’ and E indices. Variants labelled with the same letter are not significantly different (p ≤ 0.05).

The opposite was true for the fungal community, which showed a higher diversity in the root endosphere than in the rhizosphere during the fall and following spring. Overwintering did not alter either bacterial or fungal alpha diversity, regardless of rhizosphere or endosphere (Figs. 1 and 2). During the winter season, there were no significant differences between all analysed diversity indices in absorptive roots and in the rhizosphere at the three sites for both bacterial and fungal communities.

### Bacterial and fungal communities in the rhizosphere and absorptive root endosphere

The rhizosphere and root endosphere showed significant differences in bacterial and fungal communities (PERMANOVA, bacteria: F_1,55_ = 11.56, R^2^ = 0.174, p < 0.001; fungi: F_1,57_ = 37.05, R^2^ = 0.394, p < 0.001), indicating the influence of salinity on the analysed community.

The grouping in bacterial and fungal communities differed significantly for the endosphere but not for the rhizosphere (PERMANOVA, bacteria: F_2,27_ = 7.59, R^2^ = 0.360, p < 0.001; fungi: F_2,26_ = 12.28, R^2^ = 0.486, p < 0.001).

**Figure 3:**
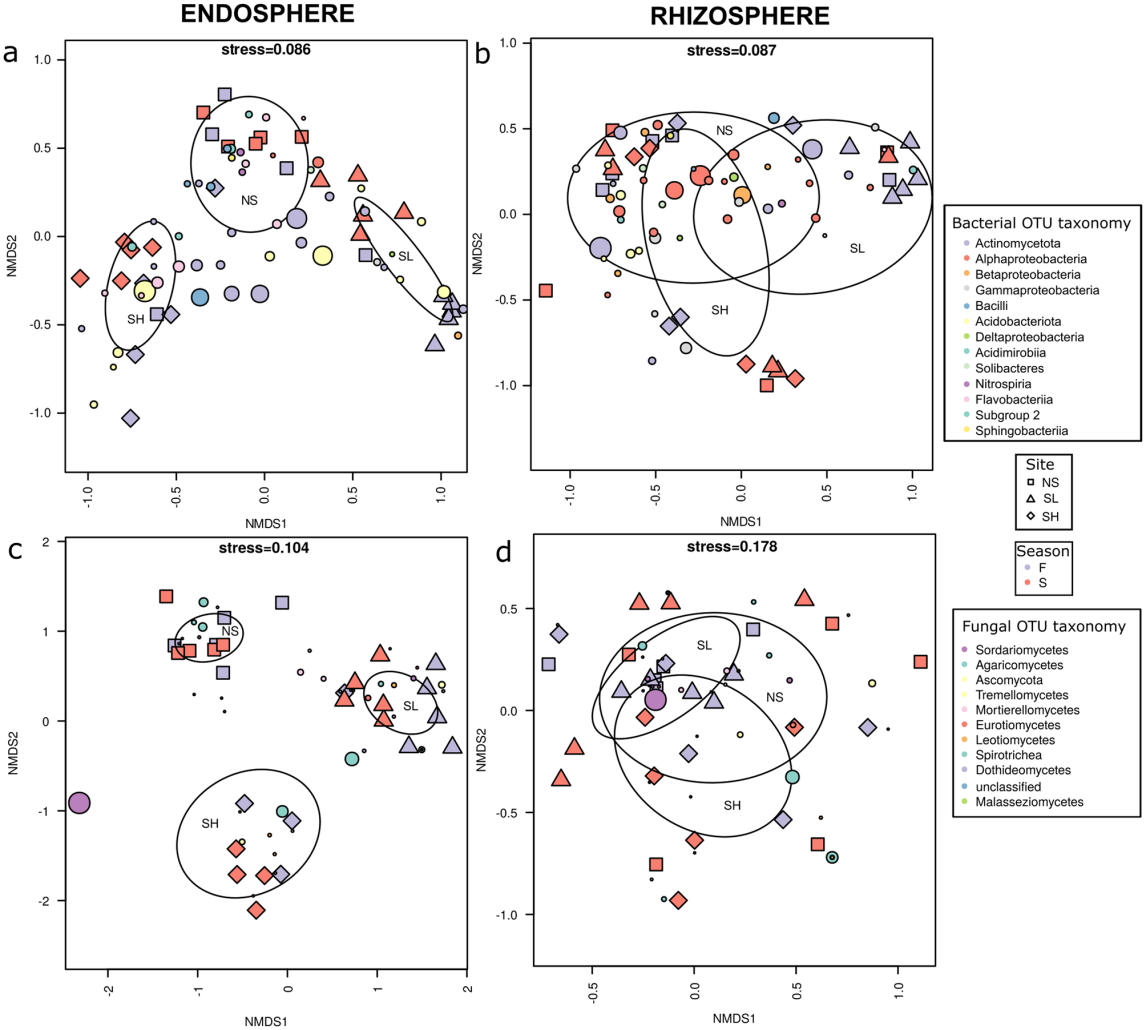
NMDS (nonmetric multidimensional scaling) analysis for (**a,b**) bacterial and (**c,d**) fungal communities. Circles represent OTUs, and their fill colour denotes consensus taxonomy at the phylum or class level. The fifty most abundant OTUs were plotted in each case. Squares, diamonds and triangles represent sites (*NS* nonsaline, *SL* saline with lower salinity, *SH* saline with higher salinity), and their fill colour indicates season (*F* fall, *S* spring). The area of the figures is proportional to the square root of EC_e_ (electrical conductivity) in (**a,c**) root endosphere and (**b,d**) rhizosphere samples.

**Figure 4:**
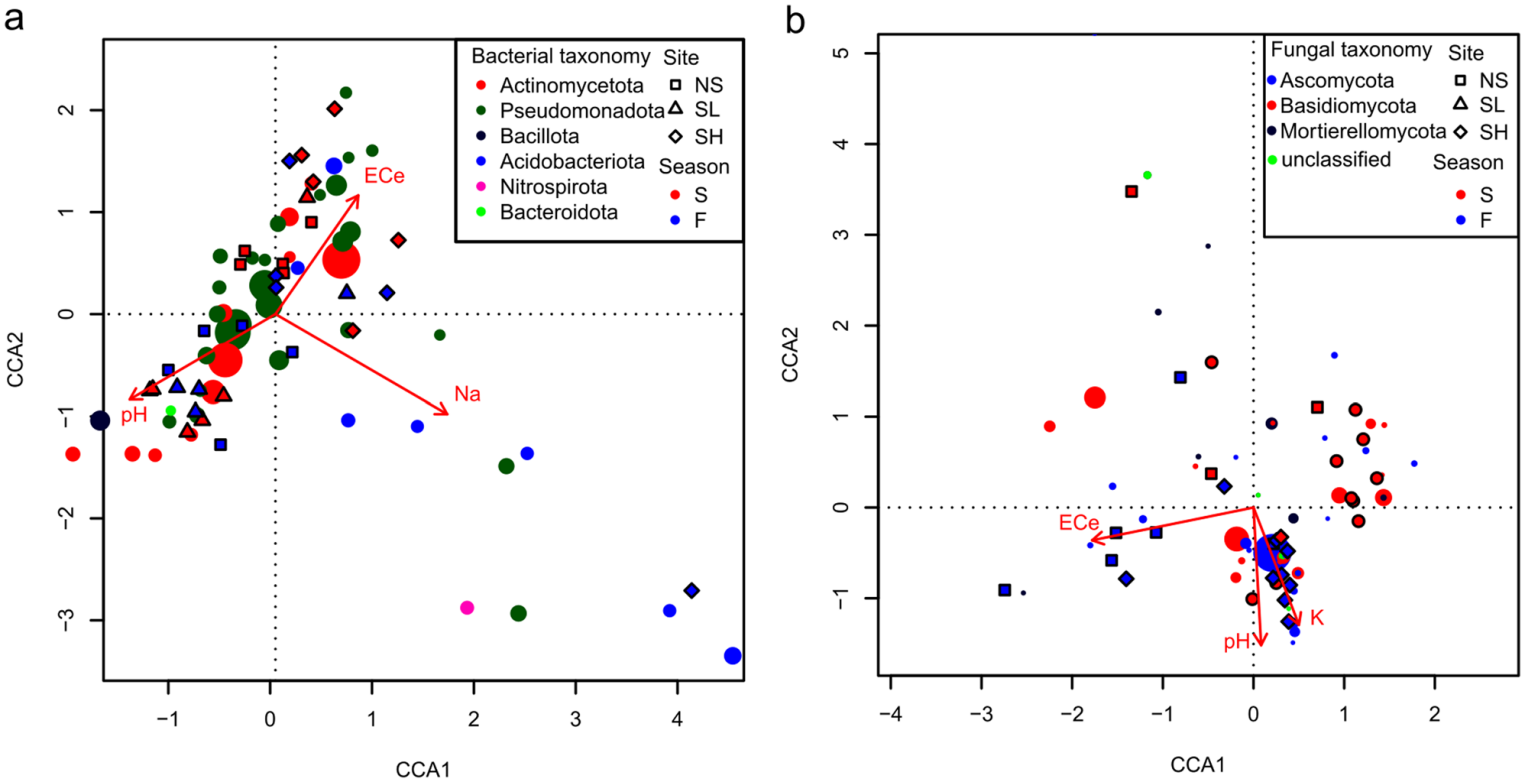
CCA (canonical correspondence analysis) for (**a**) bacterial and (**b**) fungal communities in absorptive roots of *A.* *glutinosa*. Circles represent OTUs, and their fill colour denotes consensus taxonomy at the phylum level. The fifty most abundant OTUs were plotted in each case. *NS* nonsaline, *SL* saline with lower salinity, *SH* saline with higher salinity, *F* fall, *S* spring, *EC*_*e*_ electrical conductance in saturation extract (µS × cm^−1^), *Na* concentration of sodium ions.

The effect of season drove the bacterial community structure in root samples at both saline sites (PERMANOVA, SH: F_1,8_ = 1.81, R^2^ = 0.185, p = 0.01; SL: F_1,8_ = 4.95, R^2^ = 0.382, p = 0.007) and in soil samples from SL (PERMANOVA, F_1,8_ = 2.27, R^2^ = 0.221, p = 0.02). Season also influenced the fungal community in the endosphere at the SL site (PERMANOVA, F_1,8_ = 3.61, R^2^ = 0.311, p = 0.028) (Fig. 3). According to the CCA analysis, soil salinity (EC_e_) and soil pH were significant predictors for both bacterial (Fig. 4a) and fungal (Fig. 4b) communities (permutational test (anova.cca), bacteria: F_3,26_ = 1.87, p < 0.001; fungi: F_3,25_ = 1.94, p < 0.001). Moreover, sodium and potassium ion concentrations were also significant predictors of the bacterial and fungal community structures, respectively (Fig. 4).

**Figure 5:**
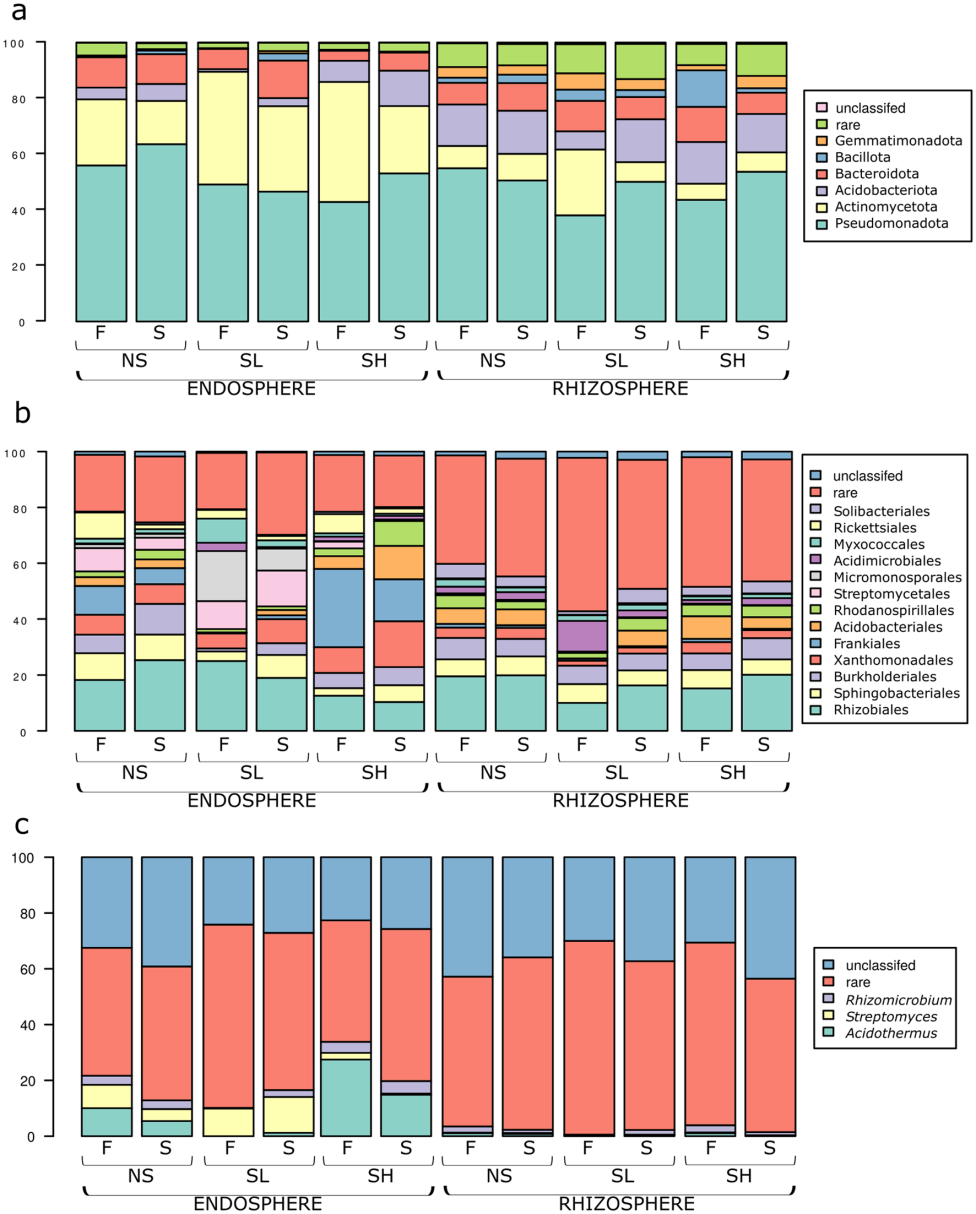
Bacterial taxonomy structure at the (**a**) phylum, (**b**) order, (**c**) or genus level in rhizosphere and root endosphere of *A.* *glutinosa* of the three sites (*NS* nonsaline, *SL* saline with lower salinity, *SH* saline with higher salinity) in two seasons (*F* fall, *S* spring). “rare” indicates taxa with less than 2% relative abundance.

The formation of specific microbial taxonomic groups depended significantly on the rhizosphere and endosphere and less on the level of salinity or season. Pseudomonadota dominated both in the endosphere and rhizosphere. Actinomycetota were more prevalent in the absorptive root endosphere (10 to 45%) than in the rhizosphere (8–38%), with the opposite trend for the Bacillota and Gemmatimonadota phyla (Fig. 5** a**). However, bacteria of lower taxonomic levels, *Streptomycetales* and *Rickettsiales,* were only present in absorptive roots, while *Solibacteriales* were present in the rhizosphere. In the endosphere, bacterial communities were characterized by the higher presence of OTUs identified as Frankiales, whereas more rare orders were identified in the rhizosphere (Fig. 5b). *Acidothermus* spp. and *Streptomyces* spp. were observed mainly in absorptive roots and rarely in the rhizosphere (Fig. 5c).

**Figure 6:**
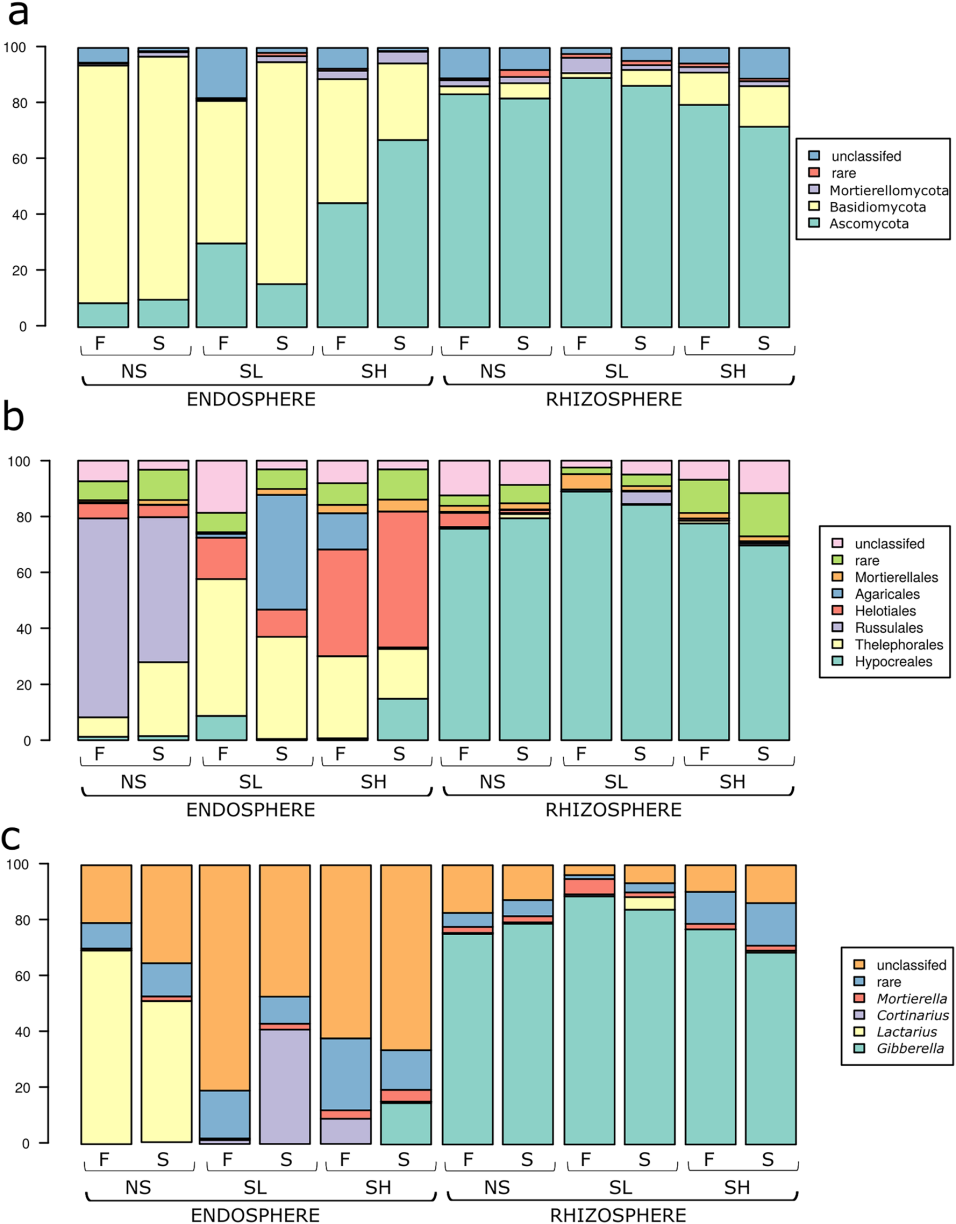
Fungal taxonomy structure at the (**a**) phylum, (**b**) order, (**c**) or genus level in rhizosphere and root endosphere of *A.* *glutinosa* of the three sites (*NS* nonsaline, *SL* saline with lower salinity, *SH* saline with higher salinity) in two seasons (*F* fall, *S* spring). “rare” indicates taxa with less than 2% relative abundance.

In terms of fungi, Basidiomycota occurred mostly in the endosphere, whereas Ascomycota occurred in the rhizosphere (Fig. 6a). Among them, Thelephorales, Russulales and Helotiales dominated in roots, and Hypocreales dominated in the rhizosphere (Fig. 6b). *Cortinarius* spp. and *Lactarius* spp. together with unclassified fungal groups dominated in root libraries, whereas *Giberella* spp. were the most frequent OTUs in rhizosphere samples (Fig. 6c).

The increase in soil salinity significantly changed the number of bacterial OTUs in root samples. The relative abundances of Acidobacteriota, Actinomycetota and Bacteroidota differed significantly between sites, with a higher presence of Actinobactecteriota at saline sites than at NS sites (Kruskal‒Wallis test, q = 0.0407). The presence of Frankiales and Acidobacteriales orders were significantly higher at SH compared to that of Streptomycetales and Micromonosporales, which were characteristic for SL. Salinity significantly influenced the presence of two main bacterial species, *Streptomyces* and *Acidothermus,* with a significantly higher abundance of the second species at the SH site (Kruskal‒Wallis test, q = 0.002) (Fig. 5b). The level of salinity also influenced the presence of fungal groups in the endosphere and not in the rhizosphere. At each site, there was a dominant fungal order: Russulales at NS, Agaricales at SL and Helotiales at SH (Fig. 6b). Microbial communities showed obvious differences during the seasons. Acidobacteriota, Bacteroidota and Bacillota had a higher presence in the fall than in early spring (endosphere-SL). Seasonal changes were noted for all bacteria in the rhizosphere (Fig. 5a), except for Bacillota (rhizosphere-SL). In the spring, the following orders were more frequent than in the previous fall: Sphingobacteriales, Burholderiales (endosphere-SL), Xanthomonadales, Acidobacteriales (endosphere-SH, endosphere-SL), Rhodanospiralles (endosphere-SH), and Acidobacteriales (rhizosphere-SL). However, Frankiales (endosphere-SH), Micromonosporalles and Myxococcales (endosphere-SH) were found more often in the fall (Fig. 5b). Fungal communities were similar during the two seasons. The only differences in occurrence were noted for Agaricales (endosphere-SH), Auriculares, Hypocreales (endosphere-SL) and Mortierellales (rhizosphere-SL) orders (fall > spring) (Fig. 6b).

### Absorptive root length and ECM morphotype colonization

During the growing season (April–October), the total lengths of absorptive root growth varied by site, ranging from 1488 cm at the NS site to 195 cm and 241 cm at the SL and SH sites, respectively. Hence, the average absorptive root growth decreased significantly from the NS site compared to the saline sites (SL and SH) (*p* ≤ 0.034, Supplementary material, Fig. 3) and, as expected, also decreased significantly towards the end of the growing season (*p* ≤ 0.024, Supplementary material, Fig. 3).

The average individual elongation of absorptive root tips by site ranged from 0.62 cm (NS) to 0.88 cm (SL) and 0.81 cm (SH). However, while the effect of salinity or season and the interaction was not significant, indicating a high variability in individual root elongation length, the average absorptive root tip length at the NS site decreased significantly during the season from 0.74 cm and 0.51 cm in the spring and summer to 0.11 cm in the fall (p ≤ 0.002, Supplementary material, Fig. 3) compared to the saline soil sites.

### Assessment of ECM morphotypes and metagenomic analysis

**Figure 7:**
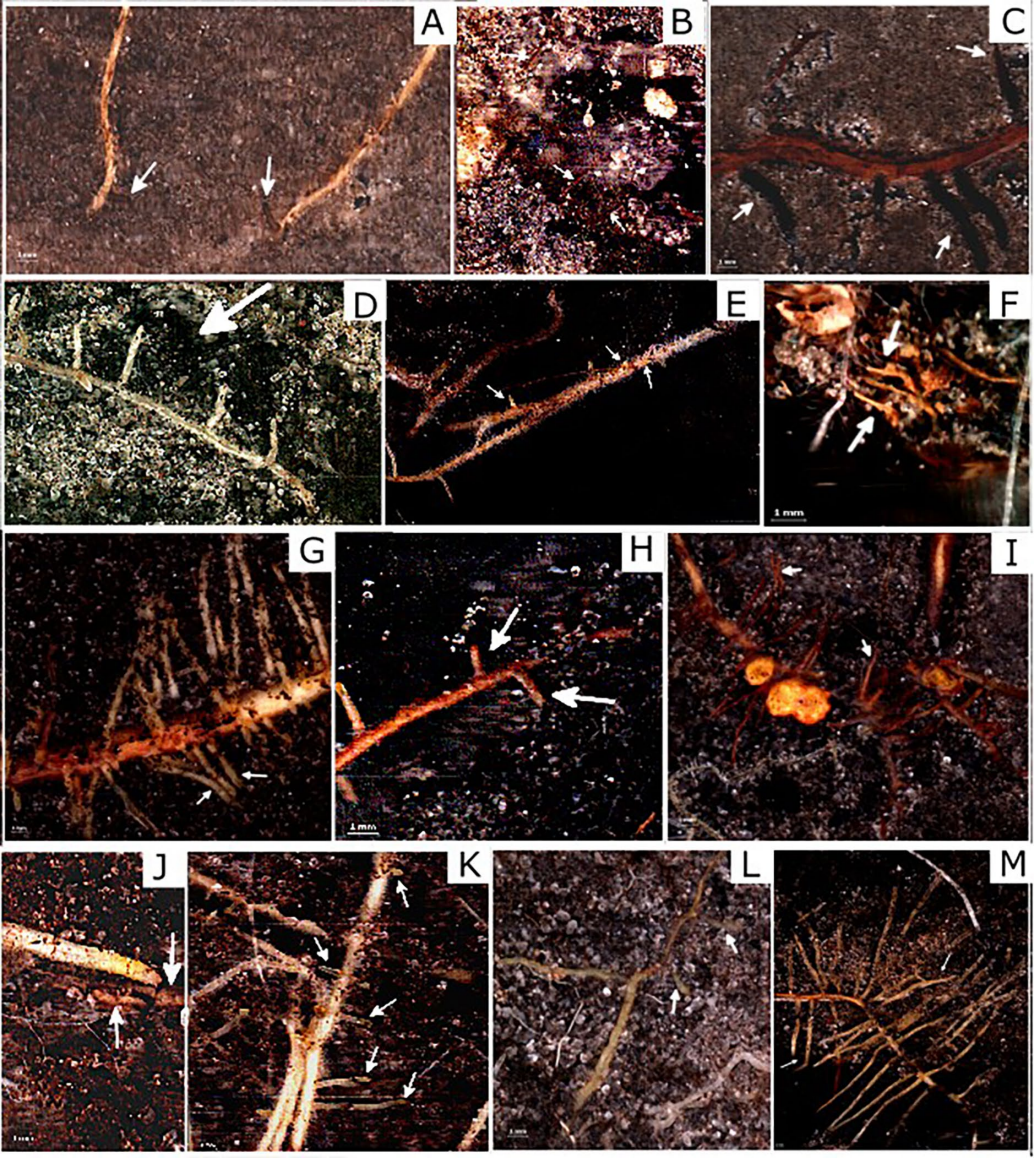
Ectomycorrhizal morphotypes identified in images of minirhizotron tubes at the three sites (*NS* nonsaline, *SL* saline with lower salinity, *SH* saline with higher salinity): (**A**) M1, (**B**) M2, (**C**) M3, (**D**) M4, (**E**) M5, (**F**) M6, (**G**) M7, (**H**) M8, (**I**) M9, (**J**) M10, (**K**) M11, (**L**) M12, (**M**) M13 (for abbreviations of morphotypes see Supplementary material, Table 2).

In total, we visually identified 13 ECM morphotypes on root tips, with 12 morphotypes present at NS, 11 at SH and 7 at SL. At all sites, we identified *Thelephora* spp., *Tomentella* spp. 2, *Cortinarius* spp., *Gyrodon* *lividus*, *Lactarius* spp. and *Tricholoma* spp. The most frequent morphotype was *Thelephora* spp. with a smooth, dark brown to black mantle, especially at the two saline sites (SL and SH) (Fig. 7; Supplementary material, Table 2). The number of root tips colonized with *Thelephora* spp. increased from spring to fall at the NS and SL sites at both soil depths. The mantle formation was the highest in spring and summer at site SH and highest at the NS site during fall. Some ECM morphotypes colonizing *Alnus* roots at the NS site were observed rarely at SL and SH, e.g., morphotype no. 7 or *Melanogaster* spp., 1–6, 1–9 root tips, respectively (Supplementary material, Table 2). The occurrence of *Russula* spp. was exclusively at the NS site (Fig. 7M; Supplementary material, Table 2). Although *Cortinarius* spp. was observed at all sites, the number of these white, woolly ECM tips decreased with increasing salinity (Fig. 7 D; Supplementary material, Table 2).

The analysis of the microbial compositions linked to the absorptive root endosphere in the spring was confirmed by the presence of most morphotypes of ECM fungi identified from the Deemy database (8 from 13 morphotypes) (marked by star symbol in Supplementary material, Table 2). The identified fungi included the genera *Cortinarius*, *Lactarius*, *Inocybe*, and *Tricholoma* and the species *Cortinarius* *alnetorum*, *Russula* *alnetorum*, *Thelephora* *alnii*, *Tomentella* *ellisii*, and *T.* *stuposa*. The identified fungi above were also active ectomycorrhizae during the growing season. In the same analysis, any DNA of fungi or only a negligible number of ectomycorrhizal nodes were observed during the growing season, i.e., *Alnirhiza* sp., *Gyrodon* sp., *Melanogaster* sp. and *Naucoria* sp., or by the presence of fruiting bodies.

## Discussion

Previous research with culture-independent methods contributed to expanding knowledge on the composition of root-associated microbial communities in both the rhizosphere and the endosphere of different plant species^25,26^. These studies allowed the holistic characterization of the root microbiota of tree species in the rhizosphere and endosphere, with a focus only on *Populus* sp. and *Cupressus* sp. (e.g.^27,28^). According to our first hypothesis, the bacterial alpha diversity was higher in the rhizosphere of absorptive roots. However, we observed that fungal diversity also showed an opposite trend, indicating that *Alnus* roots have greater selection against bacteria than against fungal endophytes. To date, the results of previous studies on bacterial alpha diversity in the rhizosphere and endosphere of trees are in agreement with our findings and show higher bacterial alpha diversity in soil than in roots (e.g.^27–29^). Unfortunately, no one has identified the role of tree root selection in changing the alpha diversity of fungal endophytes. Only Shannon’s indices indicated that roots and soil are independent of native and tropical mountain forests^29^or Acacia plantations with two different types of soil (Oxisols vs. Ultisols). Moreover, it is well known that roots can recruit beneficial microorganisms, especially under abiotic stress^30^. For example, the newest study by Oppenheimer-Shaanan et al.^30^ on the dynamic rhizosphere of C*upressus* sp. confirms that root exudation rates increase, and secreted metabolites stimulate bacterial growth under water stress. Therefore, we suggest that a higher fungal richness may have a beneficial effect on the overall condition of plants.

The lack of differences in alpha diversity between fall and spring samples might mean that overwintering did not cause bacterial death and decay but rather caused them to be dormant. Dormancy might have been caused by low temperature and by a decreased supply of carbohydrates from absorptive roots. The dormancy of woody plant species can terminate metabolism and temporarily suspend plant growth^31^. Similarly, microorganisms enter a reversible state of low metabolic activity when faced with unfavourable environmental conditions^32^. We suggest that without the supply of photosynthetic products and the release of exudates into the rhizosphere, both root endophytic and rhizosphere microorganisms in our studies seemed to have ceased their growth during the winter months, consequently resulting in an unchanged species richness. There are no studies that have analysed microbiome diversity between fall and spring that can confirm our assumption. However, studies of microbial seasonal changes in the soil of *Corylus* sp. showed a decrease in the fungal OTU richness index compared to the other three seasons when the richness increased between spring and fall^28^.

Therefore, three factors seemed influential on the relative abundance of specific bacterial and fungal taxa: the greatest differentiation can be made by its origin, the endosphere in the absorptive root and the rhizosphere, and in addition, the soil (salinity) and the season (fall and spring).

Undoubtedly, the significant effect of the source on the analysed endophytic communities can be considered selective steps of microorganisms from the soil to the rhizosphere to the plant roots because the “plant root microbiome” originates from the local soil microbial community and is shaped by the composition of tree root exudates^33^. In our study, *Alnus* allowed some microorganisms to grow into the absorptive roots, with an increased presence of bacteria belonging to the Actinomycetota phylum and Streptomycetales, Rickettsiales, and Frankiales orders; *Acidothermus* and *Streptomyces* spp. and fungi belonging to Thelephorales, Russulales, and Helotiales orders; *Lactarius* and *Cortinarius* genera. Our results are in line with a higher abundance of some bacterial taxa found in cinnamon tree roots^34^ and some taxonomic fungal groups in poplar roots than in the rhizosphere^35^. The listed root endophytes may be those specific to *Alnus* trees, which allows them to grow into absorptive roots and attract them by the constituents of plant root exudates.

Exudation of absorptive roots can attract microbial partners from the local soil community^33^, which highly depends on the physiological state of the host^36^, as well as changes in response to environmental parameters^37,38^. Most likely, plant dormancy and low temperature during winter significantly decreased the relative abundance of microorganisms in the endosphere of absorptive roots during the spring season (fall > spring, e.g., Acidobacteriota, Bacteroidota, Bacillota). However, some microbial groups were more abundant during the following spring (fall < spring, e.g., Frankiales, Agaricales, and Mortierellales), and the higher abundance of specific microbes during spring can be related to the fact that tree beneficial microorganisms are stimulated by new root elongation growth at the beginning of the growing season. One example is mutualistic symbiosis with *Frankia* spp., located within the order Frankiales, which is a striking example of successful coevolution promoting plant growth by facilitating access to scarce nutrients, particularly to nitrogenous compounds^39^.

According to our second hypothesis, the microbial community structure was driven by salinity, with significant changes only related to root endophytes. With increasing salinity, the dominant groups of bacteria and fungi significantly changed. The salinity at SH determined the presence of certain bacteria belonging to the Acidobacteriales and Frankiales orders and specific Helotiales fungi. We also found evidence on the effect of salinity on the soil microbial community structure, determining that soil salinity was a critical environmental filter, changing the relative abundance of rhizosphere taxa^40,41^ and endophytic microorganisms^19,42^. However, differences in the endophytic community structure may have been the result of the disruptive activity of salt on absorptive roots.

The average absorptive root growth, was significantly reduced with the increase in soil salinity, which we predicted in the second hypothesis, along with the decrease in the morphometric parameters of the fine roots with increasing salt concentration. In a pot experiment, Kulczyk-Skrzeszewska and Kieliszewska-Rokicka^43^ found that the highest salinity treatment (250 mM NaCl) resulted in a significant, twofold decrease in the number of poplar fine root tips compared to lower salinity levels (50 mM and 150 mM NaCl). The restricted elongation growth resulting from soil salinity not only reduced the soil volume that can be explored by absorptive roots and reduced the availability and uptake of water and essential nutrients but also diminished the supply to the aboveground parts of the host plants^44^. The negative effect of a decreased nutrient supply to the aboveground organs possibly also results in the reduced supply of assimilates with absorptive roots and symbionts, e.g., ECMs^44^, as well as the capacity of root exudation, influencing the soil microbial community in the rhizosphere^45^. These findings correspond with our observation of lower morphological diversity of ECM fungi, as well as the overall lower number of total absorptive root tips at the saline sites.

The identified ECM morphotypes, *Russula* and *Cortinarius* spp. (Supplementary material, Table 2) on absorptive *Alnus* roots in the MR images indicate an overall lower tolerance to salinity of the two genera than others, e.g., *Thelephora* and *Tomentella* spp. and confirm the sensitivity of Basidiomycetes fungi to saline soil conditions^46^. This finding confirms our third hypothesis about the formation of the ECM core and the negative effect of salinity on fungal abundance. However, some ECM species seem to cope with relatively high soil salinity concentrations and sometimes stimulate ECM growth^47^, further confirming our findings of *Tomentella* 2 spp., forming more ECM tips in saline soils than in nonsaline soil. Thelephorales constitute the core of ECM fungi in *Alnus* roots, are present in the fall and form the main ECM group in spring. However, the presence of Thelophorales was inconsistent during the growing season. Most of the ECM fungal genera identified in spring could be observed during the growing season in the MR analysis, indicating that overwintering fungi colonized emerging absorptive root tips. In addition, during the following growing season, four new ECM morphotypes appeared, colonizing several root tips of *A.* *glutinosa*.

In conclusion, the rhizosphere and endosphere of absorptive roots in the stands of *A.* *glutinosa* revealed a dynamic community structure, seemingly influenced by the changing soil environmental conditions in response to the presence of soil salinity and the growing season. In response to different salinity levels, absorptive *A.* *glutinosa* roots were associated with specific groups of bacteria, e.g., at the higher salinity site (EC_e_ = 146.7–389 μS × cm^−1^) with *Acidothermus* sp., and more diverse fungal endophytes with changing domination according to the increasing soil salinity (Russulales → Agaricales → Helotiales). Our research has shown that the use of two approaches, metagenomic amplicon analysis and in situ MR analysis, is a good solution to study the microbiomes of forest trees, especially the observation of the formed ectomycorrhizal tips. Analysis of the actual growth of absorptive roots during the growing season showed that the core formed by Thelephorales creates a basic community of ectomycorrhizal fungi, however in the future it will be needed to check the adaptation of this fungi to the saline soil environment. In the future, the groups of microorganisms we have observed should be examined in the context of creating a bioinculum that improves the growth of alder or other tree species with dual mycorrhiza in saline soil.

### Supplementary Information

Supplementary Information.

## Data Availability

Sequence files and metadata for all samples used in this study have been deposited in NCBI database under the following Bioproject numbers: root metagenome-PRJNA992246 and rhizosphere metagenome-PRJNA992247.
