# Supplementary material for: Endophytic microbiota and ectomycorrhizal structure of Alnus glutinosa Gaertn. at saline and nonsaline forest sites
Source: Sci Rep. 2023 Dec 20;13:22831. doi: 10.1038/s41598-023-49447-w (PMC10739818; doi:10.1038/s41598-023-49447-w)

Supplementary Material

Endophytic microbiota and ectomycorrhizal structure of *Alnus glutinosa* Gaertn. at saline and nonsaline forest sites

Dominika Thiem[1,2], Marc Goebel[3], Marcin Gołębiewski[2,4], Christel Baum[5], Piotr Koczorski[1], Sonia Szymańska[1,2], Katarzyna Hrynkiewicz[1,2]

[1] Department of Microbiology, Faculty of Biological and Veterinary Sciences, Nicolaus Copernicus University (NCU), Lwowska 1, PL-89-100 Torun, Poland,

[2] Centre of Modern Interdisciplinary Technologies, NCU, Wilenska 4, 87-100 Torun, Poland

[3] Department of Natural Resources and the Environment, Cornell University, 111 Fernow Hall, Ithaca, NY 14853, USA

[4] Chair of Plant Physiology and Biotechnology, Faculty of Biological and Veterinary Sciences, NCU, Lwowska 1, 87-100 Torun, Poland

[5] Soil Science, University of Rostock, Justus-von-Liebig-Weg 6, D-18059 Rostock, Germany

Corresponding author: Dominika Thiem; email: thiem@umk.p

Table 1 Averages of physicochemical soil parameters at three sites with different soil salinity (NS – nonsaline, SL – with lower salinity, SH – with higher salinity) during two seasons (fall, spring), Kruskal–Wallis tests with Dunn's test as *post hoc* comparisons (n=5 samples per site). Variants with the same letters are not significantly different ($p \leq 0.05$).

season	fall 2017			spring 2018		
parameter / site	NS	SL	SH	NS	SL	SH
EC_e (µS/cm)	41 ± 13 **b**	67.2 ± 19.5 **b**	376 ± 13**d**	27.6 ± 5.9 **a**	55.0 ± 9.5 **b**	177 ± 30.3 **c**
pH_{CaCl2}	4.9 ± 0.2 **a**	6.2 ± 0.1 **c**	5.4 ± 0.2 **b**	5.3 ± 0.3 **ab**	6.2 ± 0.1 **c**	5.0 ± 0.3 **a**
TN (%)	0.3 ± 0.1 **c**	0.1 ± 0.01 **a**	0.14 ± 0.1 **ab**	0.3 ± 0.1 **c**	0.2 ± 0.04 **ab**	0.2 ± 0.07 **bc**
TOC (%)	3.5 ± 1 **c**	1.3 ± 0.2 **a**	1.65 ± 1.1 **ab**	3.7 ± 1.0 **c**	1.8 ± 0.4 **b**	2.4 ± 0.8 **bc**
P (mg/kg)	416 **a**	369 **a**	394 **a**	390 **a**	374 **a**	343 **a**
Ca^{2+} (mg/g)	8.6 ± 0.5 **c**	6.8 ± 0.3 **b**	11.6 ± 0.5 **abc**	7.3 ± 2.4 **abc**	6.7 ± 0.2 **a**	6.0 ± 3.0 **abc**
Mg^{2+} (mg/g)	0.71 ± 0.1 **ab**	0.6 ± 0.02 **a**	0.69 ± 0.1 **b**	0.7 ± 0.05 **ab**	0.7 ± 0.03 **b**	0.6 ± 0.2 **ab**
K^+ (mg/g)	0.91 ± 0.1 **a**	0.8 ± 0.02 **a**	0.90 ± 0.1 **a**	0.7 ± 0.2 **a**	0.8 ± 0.1 **a**	0.7 ± 0.3 **a**
Na^+ (mg/g)	0.17 ± 0.1 **a**	0.2 ± 0.03 **b**	0.26 ± 0.1 **b**	0.2 ± 0.04 **ab**	0.2 ± 0.02 **ab**	0.4 ± 0.3 **ab**
moisture (%)	19 ± 6 **ab**	12.9 ± 2.4 **a**	31.7 ± 9.4 **bcd**	39.9 ± 6.0 **d**	28.1 ± 4.3 **bc**	33.1 ± 7.8 **cd**

Abbreviations: EC_e – electrical conductance in saturation extract ($\mu S \times cm^{-1}$), pH_{CaCl2} – pH measured in calcium chloride, TN – level of total nitrogen (%), TOC – level of total carbon (%), P – level of phosphorus (mg/kg)

Table 2 Number of ECM root tips according to morphotypes presented as the average from 5 analysed tubes (ave ± SE) at each test site (NS – nonsaline, SL – saline with lower salinity, SH – saline with higher salinity) and for three seasons (spring, summer, fall) at two depth levels (L1: 0-12 cm and L2: 12-24 cm).

**nr**	**description of morphotype**	**colour of mantle**	**approximate identification based on Deemy database**	**Site**	**spring**		**summer**		**fall**	
					L1	L2	L1	L2	L1	L2
M1	MC: from dark brown to black; RAM: absent; S: smooth; *R:* absent; EH: absent	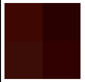	*Thelephora* sp.*	**NS**	5 ± 0.2	-	23 ± 0.5	23 ± 1.0	65 ± 3.1	33 ± 1.0
				**SL**	18 ± 1.0	5 ± 0.3	28 ± 0.9	34 ± 1.1	38 ± 1.2	57 ± 2.4
				**SH**	15 ± 0.6	12 ± 0.6	41 ± 0.9	15 ± 0.6	36 ± 0.9	13 ± 0.6
M2	MC: brown; RAM: absent; S: shiny; R: absent; EH: absent	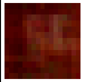	*Tomentella* sp. 1*	**NS**	-	-	-	-	-	-
				**SL**	1 ± 0.0	-	2 ± 0.1	-	1 ± 0.0	1 ± 0.1
				**SH**	-	-	-	-	-	-
M3	MC: black; RAM: absent; S: smooth; R: absent, EH: present	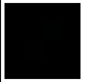	*Tomentella* sp. 2*	**NS**	1 ± 0.1	7 ± 0.5	-	4 ± 0.3	-	3 ± 0.2
				**SL**	8 ± 0.4	2 ± 0.2	1 ± 0.0	-	-	1 ± 0.1
				**SH**	5 ± 0.2	3 ± 0.1	4 ± 0.2	19 ± 1.3	7 ± 0.3	26 ± 2
M4	MC: white; RAM: absent; S: wooly; R: absent; EH: absent	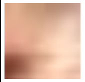	*Cortinarius* sp.*	**NS**	42 ±	13 ± 0.7	19 ±	11 ± 0.6	26 ± 0.8	15 ± 0.4
				**SL**	2 ± 0.1	1 ± 0.0	-	9 ± 0.6	-	3 ± 0.2
				**SH**	4 ± 0.2	2 ± 0.1	2 ± 0.1	-	6 ± 0.3	-
M5	MC: honey brown/golden yellow; RAM: absent; S:smooth; R: absent; EH: absent	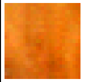	*Gyrodon lividus*	**NS**	1 ± 0.1	-	3 ± 0.2	4 ± 0.2	3 ± 0.1	1 ± 0.1
				**SL**	-	-	1 ± 0	1 ± 0.1	-	-
				**SH**	2 ± 0.1	-	1 ± 0.1	-	1 ± 0.0	-
M6	MC: light brown, creamy; RAM: absent; S: smooth; R: absent; EH: absent	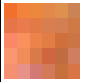	*Lactarius* sp.*	**NS**	4 ± 0.2	-	-	-	-	-
				**SL**	-	-	-	-	-	-
				**SH**	8 ± 0.6	3 ± 0.2	7 ± 0.5	-	-	-
M7	MC: olive; RAM: monopodial-pinnate; S: smooth; R: absent, EH: absent	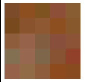	not identified	**NS**	13 ± 0.9	3 ± 0.1	13 ± 2.2	47 ± 2.7	19 ± 0.9	34 ± 1.0
				**SL**	1 ± 0.1	-	-	-	3 ± 0.1	2 ± 0.1
				**SH**	6 ± 0.3	1 ± 0.1	2 ± 0.1	-	4 ± 0.2	-
M8	MC: yellowish brown; RAM: absent; S: smooth; R: absent, EH: absent	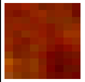	*Alpova* sp. or *Tricholoma* sp.*	**NS**	3 ± 0.2	2 ± 0.1	6 ± 0.3	3 ± 0.1	7 ± 0.3	4 ± 0.1
				**SL**	-	-	3 ± 0.1	1 ± 0.1	-	-
				**SH**	2 ± 0.2	-	7 ± 0.5	3 ± 0.2	12 ± 0.6	1 ± 0.1

M9	MC: brown; RAM: monopodial; S: smooth; R: absent, EH: absent	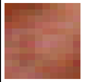	*Alnirhiza* sp.	**NS**	2 ± 0.1	-	4 ± 0.2	-	3 ± 0.2	0 ± 0.8
				**SL**	-	-	-	-	-	-
				**SH**	1 ± 0.0	-	1 ± 0.1	-	7 ± 0.5	1 ± 0.0
M10	MC: brown to red; RAM: absent; S: smooth, R: absent; EH: absent	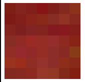	*Inocybe* sp.*	**NS**	3 ± 0.1	1 ± 0.1	6 ± 0.2	2 ± 0.1	1 ± 0.1	2 ± 0.1
				**SL**	-	-	-	-	-	-
				**SH**	-	-	9 ± 0.7	1 ± 0.1	2 ± 0.1	-
M11	MC: yellow; RAM: absent; S: shiny; R: absent; EH: absent	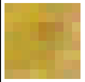	*Melanogaster* sp.	**NS**	1 ± 0.0	-	7 ± 0.3	1 ± 0.1	9 ± 0.6	2 ± 0.1
				**SL**	-	-	-	-	-	-
				**SH**	1 ± 0.0	-	-	-	-	-
M12	MC: greish; RAM: absent; S: shiny; R: absent; EH: absent	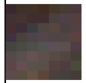	*Naucoria eschoroides*	**NS**	1 ± 0	-	-	-	-	1 ± 0.1
				**SL**	-	-	-	-	-	-
				**SH**	-	-	1 ± 0.1	0	1 ± 0.0	1 ± 0.1
M13	MC: orche; RAM: absent; S: smooth; R: absent: absent	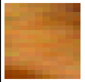	*Russula* sp.*	**NS**	25 ± 1.1	9 ± 0.6	54 +1.7	61 ± 1.9	70 ± 3.1	114 ±
				**SL**	-	-	-	-	-	-
				**SH**	-	1 ± 0.1	-	-	-	-

Abbreviations: MC – mantle colour, RAM – ramification, S – mantle surface, R – rhizomorphs, EH – emanting hyphae, * – morphotype identified in both MR and DNA amplicon analysis

Fig. 1 Average air temperature (point-line) and monthly total precipitation (bars) during 2014–2018 are in fixed proportion, 10°C corresponding to a precipitation of 20 mm, allowing the characterization of seasonal environmental conditions, i.e., drought or freeze periods (Gaussen and de Phillip 1958, Walter and Lieth 1960). Seasons are indicated by greyscale: winter (dark grey; December, January, February), spring (white; March, April, May), summer (light grey; June, July, August) and fall (black; September, October, November).

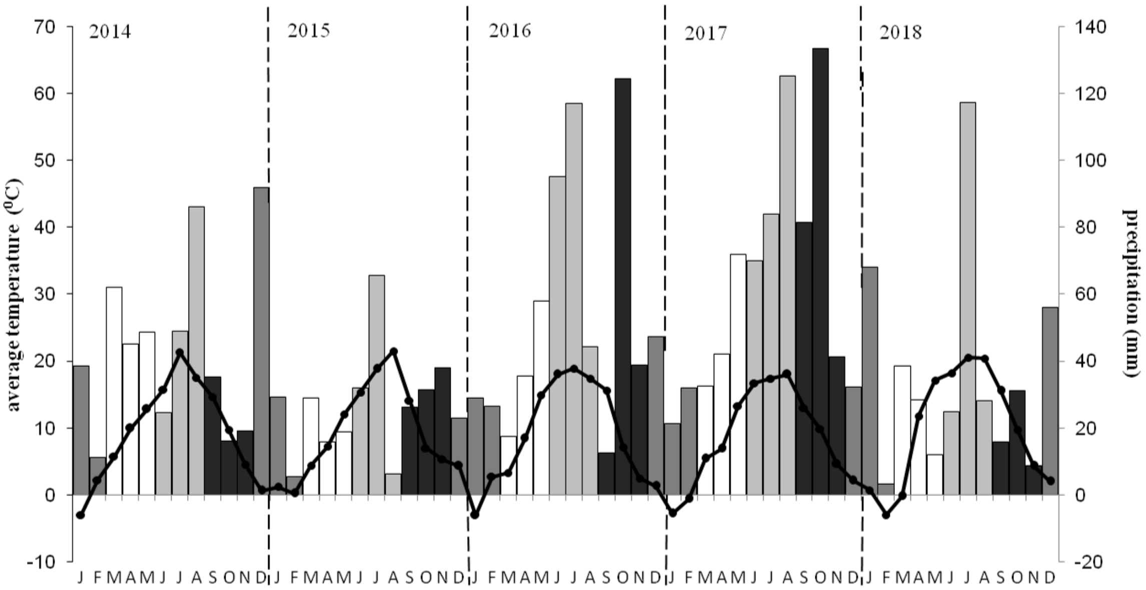

Fig. 2 Workflow of all experiments.

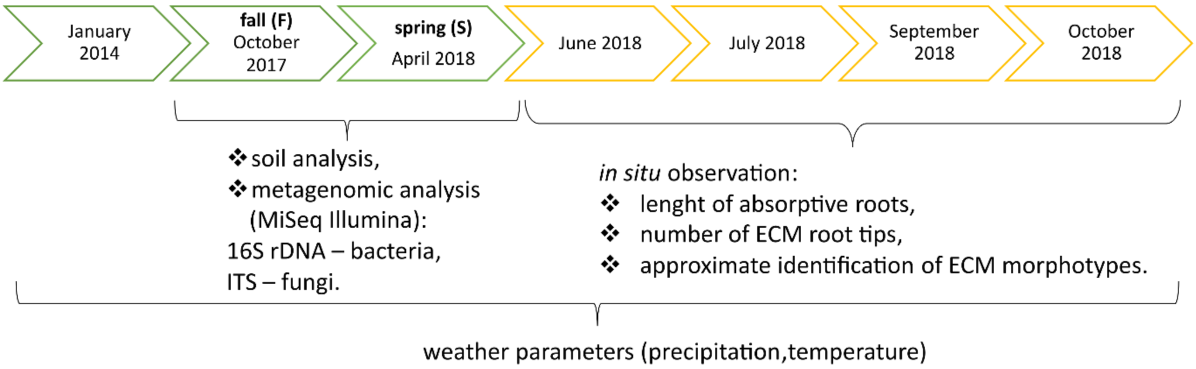

Fig. 3 Average total absorptive root length (cm) a) by site, b) by season and c) average individual absorptive root length by site and season in 2018. Absorptive roots of *A. glutinosa* Gaertn. are defined by a diameter from 0.25 mm to less than 1 mm. Different letters indicate significant differences ($p \leq 0.05$).

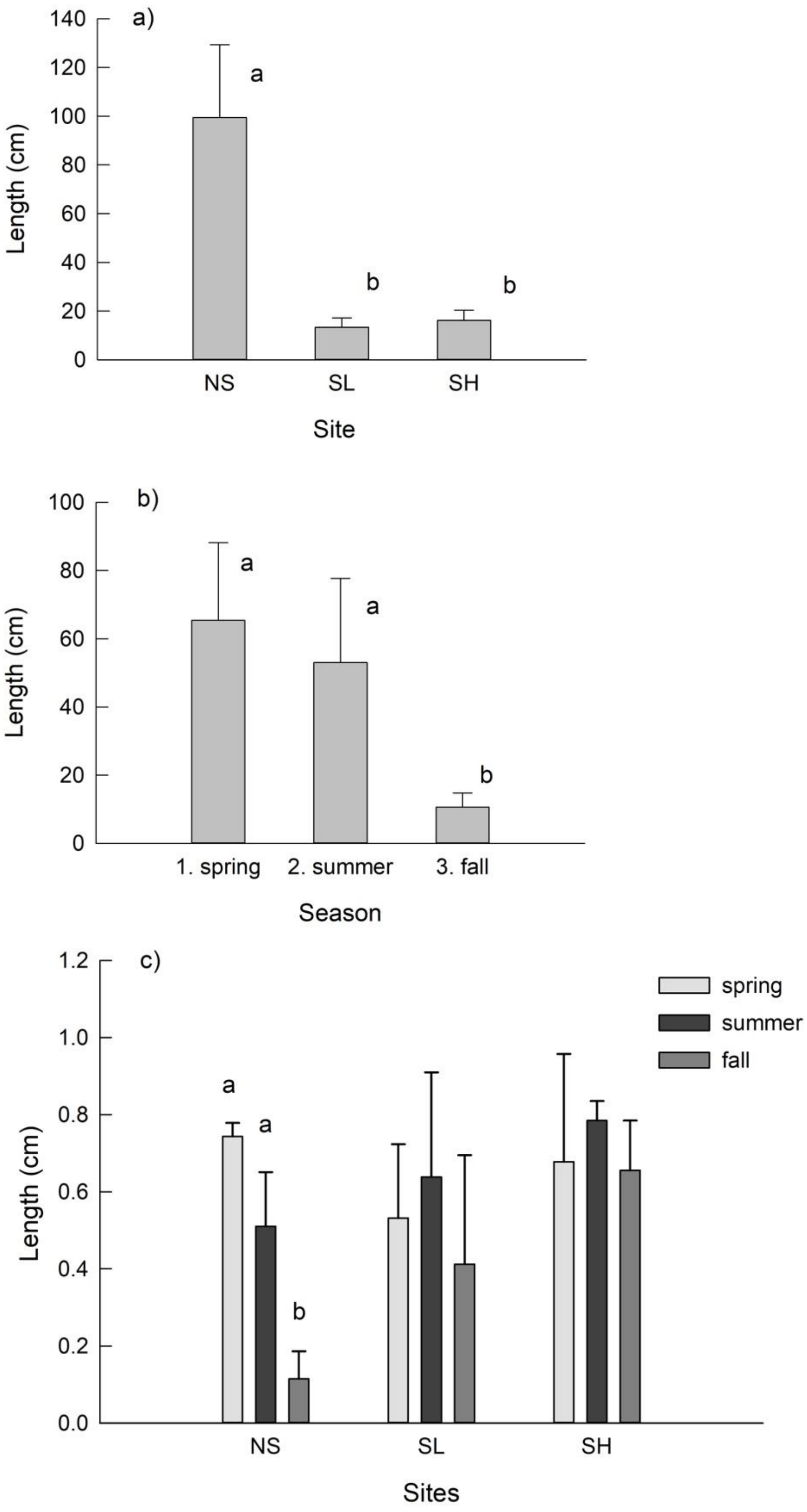

Supplement: Supplementary file 1 — Supplementary Information. [file 41598_2023_49447_MOESM1_ESM.pdf]
